# Adolescents’ primary care consultations before and after parental suicide: evidence from population-wide data

**DOI:** 10.1007/s00787-022-02095-3

**Published:** 2022-09-29

**Authors:** Rannveig K. Hart, Solveig Glestad Christiansen, Anne Reneflot, Lars Johan Hauge

**Affiliations:** Norwegian Institute of Public Health, PO Box 222 Skøyen, N-0213 Oslo, Norway

**Keywords:** Death and bereavement, Adolescence, Registry data, Mental heath, Primary care

## Abstract

Parental bereavement is associated with distress and poorer long-term outcomes among adolescents. Adolescents who lose a parent to suicide fare worse than their peers even before bereavement. Based on the current literature, we cannot distinguish such initial differences from the medium and long-term effect of parental suicide. We study the impact of parental suicide on adolescents’ General Practitioner visits for mental health or psychosocial reasons. Within-individual models account for time-invariant differences between the bereaved and non-bereaved. We investigate if effects differ from the impact of parental death from other causes, and vary with sex and socioeconomic background. Full population data on Norwegian residents aged 10–19 in the period 2006–2015 are drawn from registers (*N* = 1 405 suicide bereaved, 12 982 bereaved by other causes, and 1 182 819 non-bereaved controls). Records include data on use of health services, parental mortality, and sociodemographic characteristics of parent and child. Mental health consultations increase gradually in the quarters leading up to the parental suicide, significantly more for girls than for boys. Two years prior to bereavement, 2.4% of the subsequently suicide bereaved have a mental health consultation in any given quarter. In the year of bereavement, this increases with 6% points. Health care workers should be aware that boys are less likely to turn to their GP for support before parental bereavement from suicide.

**Supplementary Information:**

The online version contains supplementary material available at 10.1007/s00787-022-02095-3.

## Background

The loss of a parent in adolescence may leave a permanent mark. Adolescents who experience parental bereavement are found to fare worse than those who do not [1]. Parental death is associated with increased mental health problems [2–6], suicidal behavior [7, 8], somatic health problems [9], poorer school performance, behavioral problems, and increased risk taking behavior [10–13], and even increased adolescent mortality [14]. Losing a parent during childhood has also been linked to mental health problems in early adulthood [15–17]. Adolescence is a particularly sensitive period for developing mental health problems, and thus, adolescents may be especially vulnerable to experience mental health problems following parental bereavement.

The loss of a parent to suicide may be particularly detrimental. Suicide survivors often report higher levels of shame and stigma following the loss than those bereaved by other causes of death [18, 19]. While some studies conclude that parental suicide is more similar than different to other causes of death [20, 21], and also for outcomes related to mental health [19], other studies have found that parental suicide is associated with worse outcomes than parental death from other causes. This holds for depression, alcohol and substance abuse [4], anxiety [20], admission for depression [22], and also completed suicide [23–26]. For use of adolescents’ use of health services, a recent scoping review shows that research is scarce, and rarely based on longitudinal data [27].

Adoption and twin studies show substantial heritability in suicide and underlying psychiatric conditions linked to suicide [28, 29]. Adolescents whose parents die in suicide have more mental health problems, as compared to adolescents bereaved by other causes of death or non-bereaved adolescents, already prior to bereavement [20, 21]; a similar pattern is found among adults [30]. These baseline differences could confound the estimated impact of suicide bereavement itself, both as compared to parental bereavement from other causes and to non-bereaved controls.

The impact of suicide bereavement could be moderated by the sex of the adolescents. Of the few studies that have considered sex differences, the majority have found that girls are more severely impacted by parental suicide than boys. For instance, adolescent girls have a greater risk of being hospitalized for a suicide attempt shortly after parental suicide than adolescent boys [31]. In general, girls are more likely to experience higher levels of internalizing problems, stress, and fear of abandonment following parental bereavement than boys [32, 33]. A challenge with these studies is separating the overall higher prevalence of internalizing symptoms in girls, emerging in adolescence [34], from sex differences in the impact of parental loss on mental health.

The scarce literature on family socioeconomic status and parental loss suggests that resources protect: higher paternal income protected youth against attempted suicide after the death of a mother [35], and among adolescents bereaved of a parent, only those from lower socioeconomic standing experienced higher levels of high school dropout or lower grades [12, 36]. On the other hand, lower socioeconomic status is also associated with suboptimal use of health services [37], suggesting a smaller increase in consultations for those with lower socioeconomic status.

In this study, we use population-wide data to examine changes in adolescents’ General Practitioner consultations for mental health or psychosocial reasons following parental suicide. By focusing on mental health consultations in primary care, we capture mild-to-moderate mental health problems, in addition to more severe problems that require referral to specialized mental health treatment. This represents an important addition to the existing literature that has focused on severe mental health problems. The main purpose of this study is threefold. First, we examine the impact of suicide bereavement on mental health consultations; next, we examine whether there exists a unique burden of suicide. Finally, we examine if the impact of suicide bereavement varies by sex and socioeconomic background. Throughout these analyses, we account for underlying differences between the groups using within-individual (fixed effects) models.

## Methods

### Data and variables

The study population includes all adolescents aged 10–19 years from 2006 to 2015 in Norway. Capping the sample at 19 years is arguably low [38], but secures that most adolescents still lives with their parents also in the Norwegian context where age when leaving the parental home is comparatively low [39]. We test whether the results are sensitive to this restriction. Information is drawn from multiple registers, as outlined below, linked using a unique (encrypted) personal identifier. The personal identifier also allows us to link children to their parents (biological or adopted). Linkages are independent of whether parent and child live together, meaning that children, e.g., will be linked to a non-resident father, but not to a resident step-father. We include children who have immigrated themselves, or whose parents have immigrated, as long as the child and at least one parent have a personal identifier. We conduct robustness tests excluding immigrants and children of immigrants, to test if a distinct pattern emerges in the majority population.

Date and cause of death are derived from the Norwegian Cause of Death Registry. Based on the ICD-10 diagnostic system, we distinguish between parental death by suicide (X60-X84, Y87.0) and all other causes. We exclude children who experienced parental death from any cause prior to the observation period, so that our focal event will be the first parental loss. Information on loss of a second parent, either in the same quarter as the first or subsequently, is not included in our data or model.

For the multivariate analyses, we construct person quarters, i.e., four records per year a person is observed, each covering 3 months. Individuals are observed for a maximum of 7 years, and the observation time before and after parental death varies between individuals, thus resulting in an unbalanced sample. Our statistical model handles this unbalanced sample well. However, it requires at least one measurement point before parental death. Thus, individuals who experience parental death in the first quarter they are observed are excluded. Observations further than 5 years before or after parental death are rare and were deleted to avoid that the tails of the duration variable to be imprecisely estimated. The study sample included 1 405 adolescents bereaved by suicide, 12 982 adolescents bereaved by other causes, and 1 182 819 non-bereaved controls.

The outcome variable, mental health consultations, is the quarterly probability of at least one in-person consultation to the General Practitioner (GP) for mental health symptoms or diagnoses (P01-P99) or psychosocial concern (Z01-Z29) according to the International Classification of Primary Care (ICPC-2). Z-codes include a range of social concerns; among these, problems related to the illness of parent (Z22) or loss of parent (Z23) are highly relevant in our context. Data on use of primary health care services were obtained from the national database for the reimbursement of health expenses (KUHR).

We assess heterogeneous effects by the adolescent’s sex (male = 0, female = 1) and parent’s educational attainment (none higher degree = 0, at least one higher = 1). Data on parental educational attainment are derived from the National Educational Data Base (NUDB). Information on sex is obtained from the Central Population Register.

### Statistical methods

The current literature suggests that there may be differences in mental health between those who will subsequently lose a parent to suicide and those who will not, prior to the event. If so, a comparison of mental health consultations among those who have lost a parent to those who have not could be upwardly biased as a measure of the impact of parental suicide on mental health consultations. Such confounding is handled through the estimation of a within-individual (fixed effects) model. Non-bereaved controls contribute to the estimation of the period and age effects and increase precision [40].

We estimate within-individual (fixed effects) linear probability models, with the following regression equation:

$$y_{i,t } = \alpha_{i} + \mathop \sum \limits_{d = - 5}^{5} \beta_{d} x_{d, i.t} + \mathop \sum \limits_{a = 10}^{19} \beta_{a} x_{a, i.t} + \mathop \sum \limits_{y = 2007}^{2015} \beta_{y} x_{y, i.t} + \varepsilon_{i,t} .$$

$\sum\nolimits_{d = - 5}^{5} {x_{d, i.t} }$ is a set of dummy variables indicating the duration to/from parental death in years. For instance, the dummy variable ${X}_{t=2}$ takes 1 for records 2 years after parental death, otherwise zero. The year before parental death (d-1) is the omitted reference category. Non-bereaved controls have zero on all duration dummies. Standard errors are clustered at the individual.

We include control variables to isolate the effect of duration from parental death from changes in age and period time. $\sum\nolimits_{a = 10}^{19} {x_{a, i.t} }$ denotes dummy variables for each age from 10 to 19 years, taking 13 as the omitted reference category. $x_{{\text{y}}}$ are dummy variables for calendar year, with 2006 as the omitted reference category. $\alpha_{{\text{i}}}$ are individual fixed effects (intercepts) that capture any time-constant characteristics of the individual that may impact consultation frequency. With individual fixed effects, controls for time-invariant individual characteristics are redundant.

### Subsample stratification

To test hypotheses of differential effects by the child’s sex and parents’ educational attainment, we stratify the sample by these variables. To test formally whether effects vary between subsamples, we add an interaction term between duration and the stratifying variable in a pooled model (results in Table 2, column 3).

### Sensitivity analysis

We test whether our results are sensitive to two sample restrictions: First, we expand the sample by including young adults up to age 24. Second, we restrict the sample by excluding foreign-born children and children of foreign-born parents.

To test to what extent consultations for mental health and/or psychosocial concern drive our results, we estimate effects on two additional outcome variables:

- a variable excluding psychosocial presentations, taking 1 for any consultation with mental health symptoms and diagnoses (chapter P), otherwise 0
- a variable indicating psychosocial presentations only, taking 1 for any consultation with psychosocial presentations (chapter Z), otherwise 0.

The loss of a spouse to suicide is found to increase the risk of some physical diagnoses, indicating a somatising component [41]. To test for somatization, we construct an outcome variable taking 1 for those who have at least one consultation neither in chapter P nor Z, otherwise 0.

## Results

### Descriptive results

Table 1 shows descriptive statistics for the included covariates and stratifying variables, for the suicide bereaved, adolescents bereaved by other causes and the non-bereaved. For the bereaved, mental health consultations are measured 2 years prior to parental death. Panel A shows that the percentage with a mental health consultation in any given quarter is highest among those bereaved by other causes (3.0%), followed by those bereaved from suicide (2.4%), and lowest among the non-bereaved (2.0%). Across groups, about a fifth of all adolescents attended their GP for any cause (not mental health) in any given quarter.

**Table 1:** Descriptive statistics. Means and standard deviations by subsample

	Adolescents bereaved from suicide		Adolescents bereaved from other causes		Non-bereaved controls		Total	
Panel A (unit: person quarters)	Mean	(S.D.)	Mean	(S.D.)	Mean	(S.D.)	Mean	(S.D.)
Mental health consultation GP	0.024	(0.154)	0.030	(0.170)	0.020	(0.141)	0.0205	(0.142)
Psychosocial consultation GP	0.027	(0.162)	0.031	(0.172)	0.022	(0.145)	0.0217	(0.146)
Any consultation with GP	0.216	(0.412)	0.219	(0.414)	0.224	(0.417)	0.224	(0.417)
Duration from parental death (years)	− 0.572	(2.432)	− 0.670	(2.420)				
Freq. person quarters	34 374		309 222		25 116 542		25 460 138	
% Of all person quarters in sample	0.1		1.2		98.7		100.0	
Panel B (unit: persons)	Freq	%	Freq	%	Freq	%	Freq	%
Sex of adolescent								
Male	699	49.8	6 618	51.0	606 039	51.2	613 356	51.2
Female	706	50.3	6 364	49.0	576 780	48.8	583 850	48.8
Parental education								
None higher education	798	56.8	8 040	61.9	598 126	50.6	606 964	50.7
At least one higher education	607	43.2	4 942	38.1	584 693	49.4	590 242	49.3
Sex of deceased parent								
Male	978	69.6	8 903	68.6				
Female	427	30.4	4 079	31.4				
	Mean	(S.D.)	Mean	(S.D.)				
Parental age at death	45.52	(6.590)	50.07	(8.191)				
Freq. persons	1 405		12 982		1 182 819		1 197 206	
% Of all persons in sample	0.1		1.1		98.8		100.0	

Subsamples are defined by whether and how the adolescent ever experienced a parental death in the observation period. For bereaved adolescents, consultations are measured 2 years before parental death

Turning to Panel B, we see that girls are somewhat overrepresented in the suicide bereaved group (50.3%), compared to the two other groups (49.0% for bereaved by other causes and 48.8% of the non-bereaved). For suicide, 69.6% of bereaved children lost their father, and 30.4% lost their mother; the distribution is very similar for bereavement from other causes.

**Fig. 1:**
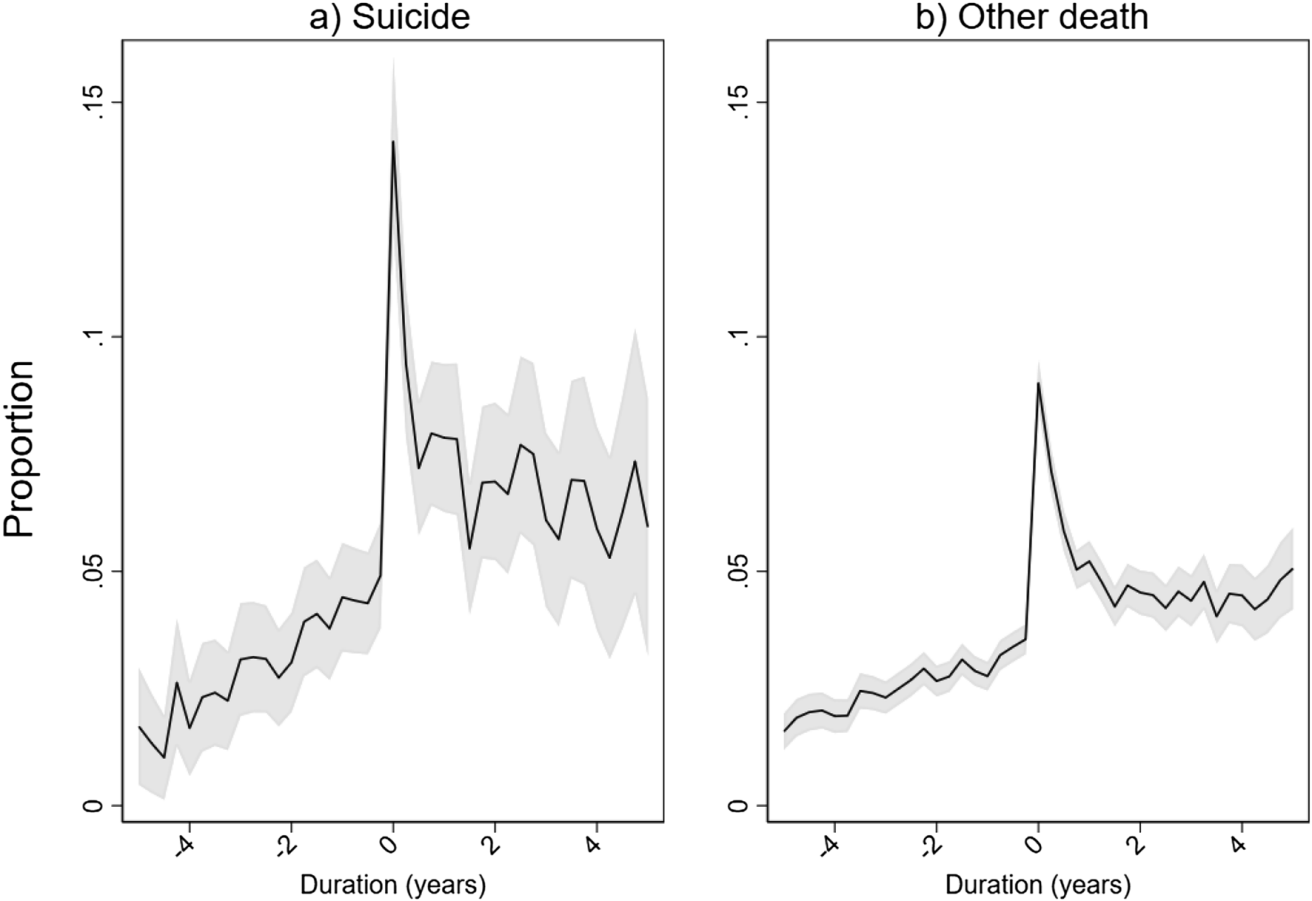
Quarterly proportion with primary care mental health consultation, by duration from time of parental death. Parental suicide sample, *N* = 34 374 person quarters, and parental bereavement from other causes (*N* = 309 222). Shaded areas give 95% confidence intervals

Figure 1a shows how the quarterly probability of a mental health consultation varies by duration to parental bereavement by suicide. The probability rises gradually up to the year of parental death, at which point it peaks at about 0.14. In the years following parental death, the probability of a mental health consultation stabilizes at a higher level than before parental suicide. A similar pattern is found for all parental deaths, at a somewhat lower level (Fig. 1b). This indicates that duration to parental death may indeed affect consultation frequency.

When duration to parental bereavement changes, so does the period time and the age of the adolescent. We found a steep and near linear increase in consultation frequency with age, and also an increase over period (Supporting Information Figure A1, Panel A). The same pattern emerges for adolescent bereaved by other causes of deaths (Panel B) and the non-bereaved (Panel C). This underlines the need to estimate multivariate models, that hold age and period time constant, to isolate the effect of duration from parental death.

### Main regression results

**Fig. 2:**
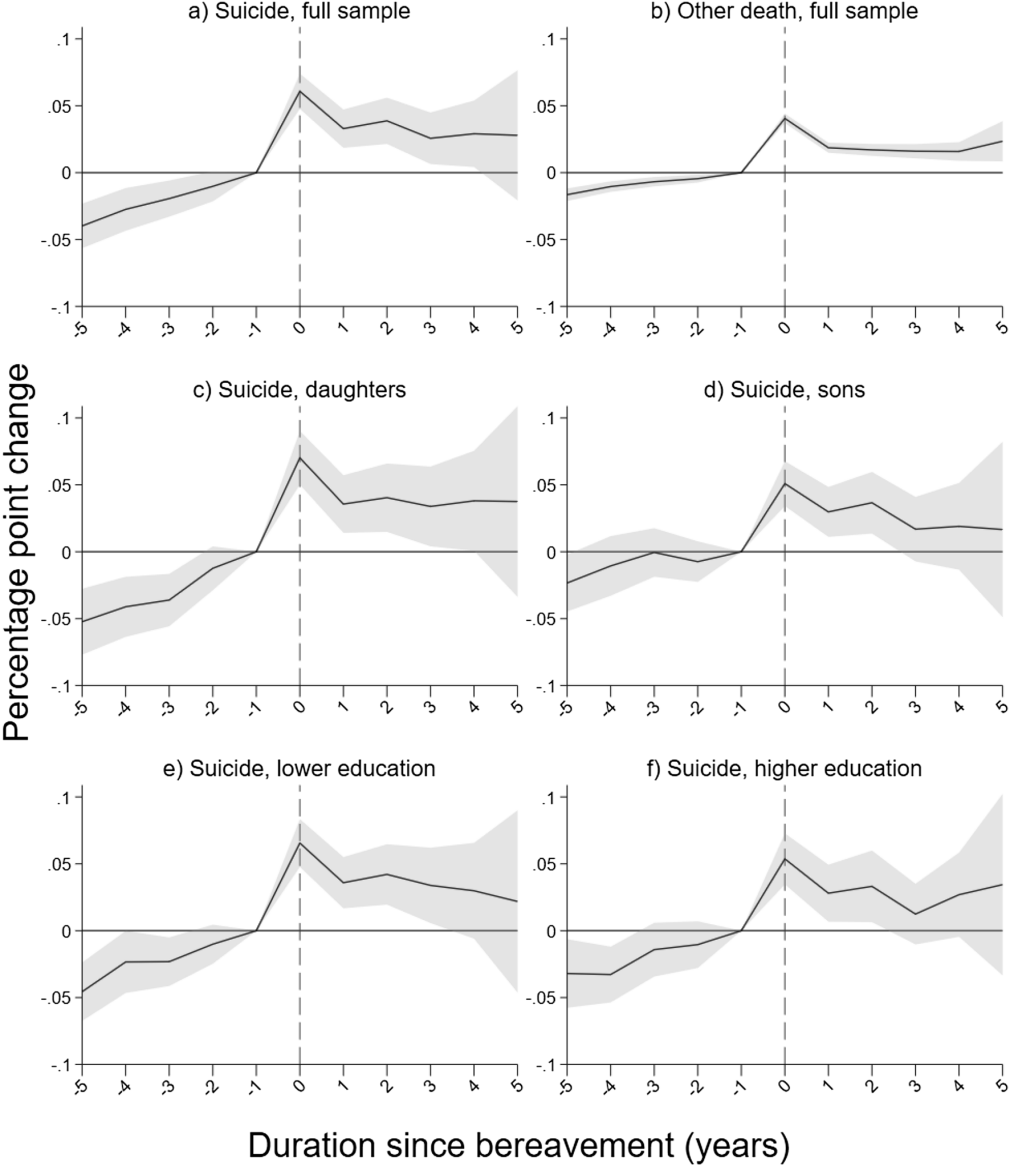
Effect of parental death on quarterly probability of primary care mental health consultation. Point estimates (full lines, see also Table 2) with 95% confidence intervals (shaded areas). Estimates are controlled for dummies for age (1 year categories), dummies for period time (1 year categories), and individual fixed effects

Taking the year prior to bereavement as the reference year, we estimate how the probability of a consultation changes, year by year, in the 4 years leading up to bereavement, the year of bereavement itself and in the 5 subsequent years. Parameter estimates are shown in Table 2 and Fig. 2. For bereavement by suicide (Panel A, column 1 and Fig. 2a), consultation frequency increases gradually up to the year of bereavement. In the year of bereavement, the probability of a consultation is 6 percentage points higher than the year before. Five years after bereavement, consultations frequencies have leveled off to 3 percentage points higher than in the reference year.

**Table 2:** Effect of duration from parental bereavement on the quarterly probability of primary care mental health consultation

Panel A	(1) Suicide, full sample			(2) Other death, full sample			(3) All deaths, full sample: suicide*		
− 5	− 0.04	(0.01)	***	− 0.02	(0.00)	***	− 0.02	(0.01)	**
− 4	− 0.03	(0.01)	***	− 0.01	(0.00)	***	− 0.02	(0.01)	*
− 3	− 0.02	(0.01)	**	− 0.01	(0.00)	***	− 0.01	(0.01)	+
− 2	− 0.01	(0.01)	+	0.00	(0.00)	**	− 0.01	(0.01)	
− 1 (ref.)									
0	0.06	(0.01)	***	0.04	(0.00)	***	0.02	(0.01)	**
1	0.03	(0.01)	***	0.02	(0.00)	***	0.01	(0.01)	+
2	0.04	(0.01)	***	0.02	(0.00)	***	0.02	(0.01)	*
3	0.03	(0.01)	**	0.02	(0.00)	***	0.01	(0.01)	
4	0.03	(0.01)	*	0.02	(0.00)	***	0.01	(0.01)	
5	0.03	(0.02)		0.02	(0.01)	**	0.00	(0.03)	
N	17 607 502			17 797 934			17 819 725		

Panel B	(1) Suicide, female			(2) Suicide, male			(3) Suicide, full sample: female*		
− 5	− 0.05	(0.01)	***	− 0.02	(0.01)	*	− 0.05	(0.02)	**
− 4	− 0.04	(0.01)	***	− 0.01	(0.01)		− 0.04	(0.02)	**
− 3	− 0.04	(0.01)	***	0.00	(0.01)		− 0.04	(0.01)	**
− 2	− 0.01	(0.01)		− 0.01	(0.01)		− 0.01	(0.01)	
− 1 (ref.)									
0	0.07	(0.01)	***	0.05	(0.01)	***	0.02	(0.01)	+
1	0.04	(0.01)	**	0.03	(0.01)	**	0.01	(0.01)	
2	0.04	(0.01)	**	0.04	(0.01)	**	0.02	(0.02)	
3	0.03	(0.02)	*	0.02	(0.01)		0.03	(0.02)	+
4	0.04	(0.02)	*	0.02	(0.02)		0.04	(0.03)	
5	0.04	(0.04)		0.02	(0.03)		0.04	(0.05)	
N	8 566 956			9 040 546			17 607 502		

Panel C	(1) Suicide, lower education			(2) Suicide, higher education			(3) Suicide, full sample: higher education*		
− 5	− 0.05	(0.01)	***	− 0.03	(0.01)	*	0.02	(0.02)	
− 4	− 0.02	(0.01)	*	− 0.03	(0.01)	**	− 0.01	(0.02)	
− 3	− 0.02	(0.01)	*	− 0.01	(0.01)		0.01	(0.01)	
− 2	− 0.01	(0.01)		− 0.01	(0.01)		0.00	(0.01)	
− 1 (ref.)									
0	0.07	(0.01)	***	0.05	(0.01)	***	− 0.01	(0.01)	
1	0.04	(0.01)	***	0.03	(0.01)	**	− 0.01	(0.01)	
2	0.04	(0.01)	***	0.03	(0.01)	*	− 0.01	(0.02)	
3	0.03	(0.01)	*	0.01	(0.01)		− 0.02	(0.02)	
4	0.03	(0.02)		0.03	(0.02)	+	− 0.01	(0.02)	
5	0.02	(0.03)		0.03	(0.03)		0.01	(0.05)	
N	9 219 021			8 388 481			17 607 502		

Parameter estimates and standard errors. Estimates are controlled for dummies for age (one year categories), dummies for period time (one year categories), and individual fixed effects. Column 3 shows estimates for the interaction term between the stratifying variable and duration to bereavement. ***p < 0.001, **p < 0.01, *p < 0.05, + p < 0.1

Turning to bereavement from other causes of death (Panel A column 2, and Fig. 2b), the point estimates show a similar pattern: a gradual increase in the years before bereavement, a peak in the year of bereavement, and then leveling off at a slightly higher level than in the reference year. However, the estimates are consistently smaller in this sample. Interaction terms between duration dummies and cause of death, estimated in a joint model, show a significantly sharper pre-bereavement increase and a higher peak around bereavement in the suicide sample (Table 2, Panel A, column 3).

### Subsample stratification: sex of adolescent and socioeconomic background

To investigate if boys and girls are impacted differently by parental suicide, we stratified our analysis by adolescent sex (Table 2, Panel B). The increase in consultation frequency in the years before is bereavement substantially larger among girls (column 1) than among boys (column 2). When tested in a joint model, the difference is statistically significant (column 3). The peak in the bereavement year and the subsequent leveling off is also at a higher level among girls than among boys, and some of the interaction estimates border statistical significance (*p* < 0.1).

We also tested whether the effects varied with parental education (Table 2, Panel C and Fig. 2e and f). There is a slight tendency of larger point estimates in the lower educated sample (column 1), but the difference is not statistically significant (column 3).

### Sensitivity tests: alternate samples and outcomes

Our first two sensitivity tests regard sample restrictions. Results are shown in Panel A of Fig. A2 and Table A1. Neither limiting the sample to adolescents with majority background, nor extending the sample up to age 24, qualitatively changes the results.

Sensitivity tests for alternate outcomes are shown in Panel B of Figure A2 and Table A1. Excluding psychosocial presentations from the outcome and running the model on mental health visits only yields a very similar pattern to the main results (left panel and column 1). When psychosocial presentations are analyzed alone (mid panel and column 2), a small peak emerges around the parental death, but no effects emerge in other years. Thus, our results are mainly driven by mental health visits, with psychosocial causes contributing to the peak in the year of parental bereavement. Finally, we find no effects on primary care visits when mental health and psychosocial visits are excluded (right panel and column 3). Thus, there is no evidence of somatization. This holds also when analyzed separately for boys and girls (results available upon request). Thus, more somatization among boys cannot explain their smaller increase in mental health consultations.

## Discussion

This study casts new light on the impact of parental bereavement to suicide in adolescence on mental health. We estimated time-to-event models on full population data on mental health consultations in primary health care, controlling for unobservable individual characteristics constant across time. Our contribution to the literature is threefold. First, we establish that the more frequent mental health consultations among suicide bereaved adolescents are at least in part a causal effect of a traumatic experience. The increase starts before the time of parental death, indicating that the time leading up to parental suicide is also a stressful period for the adolescent. While previous studies have tended to focus on severe mental health problems, we show that effects go beyond this. Adolescents bereaved by parental suicide also experience mild-to-moderate mental health problems treated in primary care. Our finding is in line with the current literature showing that adolescents bereaved by suicide have poorer mental health outcomes both pre- and post-loss [4, 20–22, 42, 43]. We expand the literature by controlling for differences between the bereaved and the non-bereaved present already prior to bereavement, and getting a precise description of how these differences evolve with duration to and from parental death.

Second, we compare the effect of bereavement by parental suicide to bereavement of a parent due to other causes. To the extent that those who lose a parent to suicide are more prone to have underlying mental health problems, differential levels of consultations in the years following bereavement may wrongly be interpreted as a more negative impact of parental suicide than other parental bereavement. Our results show that also when we account for initial differences, the burden of suicide is still different from losing a parent to other causes. This is in line with the literature finding that parental suicide has more severe consequences than other parental bereavement [4, 20, 22–25].

Third, our results cast new light on gendered patterns of suicide bereavement. Our results show that girls have substantially and significantly larger increases in mental health consultations in the years leading up to parental suicide than do boys, and there is also a tendency of a higher peak in the year of bereavement and subsequently among girls. This is in line with the current literature, which shows that girls experience higher levels of internalizing problems and stress than boys following parental bereavement in general [32, 33] and parental suicide [31] in particular. Our findings suggest that gender differentials in the years leading up to parental suicide could suggest that girls’ mental health deteriorate more in those years, and/or reflect different help-seeking patterns between suicide bereaved girls and boys. Compared to boys, girls are more likely to seek help for their mental health problem, and face fewer barriers when they *do* seek help from their GP [44]. Our findings raise the question of whether male adolescents receive adequate health care when their parents are at high risk of suicide.

Finally, we found no substantial or significant differences in the effect of suicide bereavement between children of higher educated and lower educated parents. In contrast, previous studies have suggested more detrimental effects for children of parents with lower socioeconomic status [12, 35, 36]. The different results could be related to children of lower educated parents being less likely to seek help for mental health symptoms [37].

As for clinical and policy implications, our findings suggest that the GP has an important role in providing care for suicide bereaved adolescents. Timely help in primary care may prevent the development of more severe mental health problems. Several studies of suicide bereaved young people have indicated a substantial unmet need for professional help [45]. GPs may adopt a more proactive role toward suicide bereaved boys to ensure that their health care needs are adequately met. Finally, when an adolescent with a severely mentally ill parent increases their GP mental health presentations, this should prompt outreach to the parent to screen them for suicide risk.

### Limitations

Some inherent limitations to our analytical approach and data should be noted. First, observed changes in GP visits reflect both changes in health and changes in use of health care, given health [46]. These two contributions could not be empirically separated from each other. Future research could complement our findings by estimating effects on a richer range of outcomes. Also, in-depth qualitative studies of support systems in this special grieving process would be valuable, particularly to understand whether boys are adequately met by the primary care system. Despite these limitations, we note that data from administrative and health registers have strengths of particular importance for this topic and study design. The large sample size allows us to estimate a data demanding model for a very rare event, getting us closer to an understanding of causal mechanisms. Drawing data from registers ensures a representative sample, eliminates recall bias, and ensures high validity of both predictors and outcomes.

## Conclusion

The increase in GP consultations for mental health problems following parental suicide indicates that this kind of loss causes considerable distress among adolescents. Our findings confirm that adolescents of parents that die by suicide are more vulnerable from the outset, but this vulnerability does not explain the increase in use of health services leading up to and following parental bereavement.

Timely and adequate help for mental health problems may alleviate the adolescent’s current mental health problems, and it may prevent future problems. Particularly, health care workers should be aware that boys may be less likely to turn to primary care providers for help in the years leading up to parental suicide.

### Supplementary Information

Below is the link to the electronic supplementary material.

Supplementary file1 (DOCX 282 KB)
