# Supplementary material for: Adolescents’ primary care consultations before and after parental suicide: evidence from population-wide data
Source: Eur Child Adolesc Psychiatry. 2022 Sep 29;32(12):2453–62. doi: 10.1007/s00787-022-02095-3 (PMC10682049; doi:10.1007/s00787-022-02095-3)
Supplement: Supplementary file 1 — Supplementary file1 (DOCX 282 KB) [file 787_2022_2095_MOESM1_ESM.docx]

**Figure A.1: Quarterly proportion with primary care mental health consultation, by duration from time of parental death, age and calendar year**. Parental death by suicide, N= 34 374 person quarters. Parental death to causes other than suicide, N= 309 222 person quarters. Control adolescents (not bereaved in the observation period), N=25 460 138 person quarters. Shaded areas give 95% confidence intervals. **
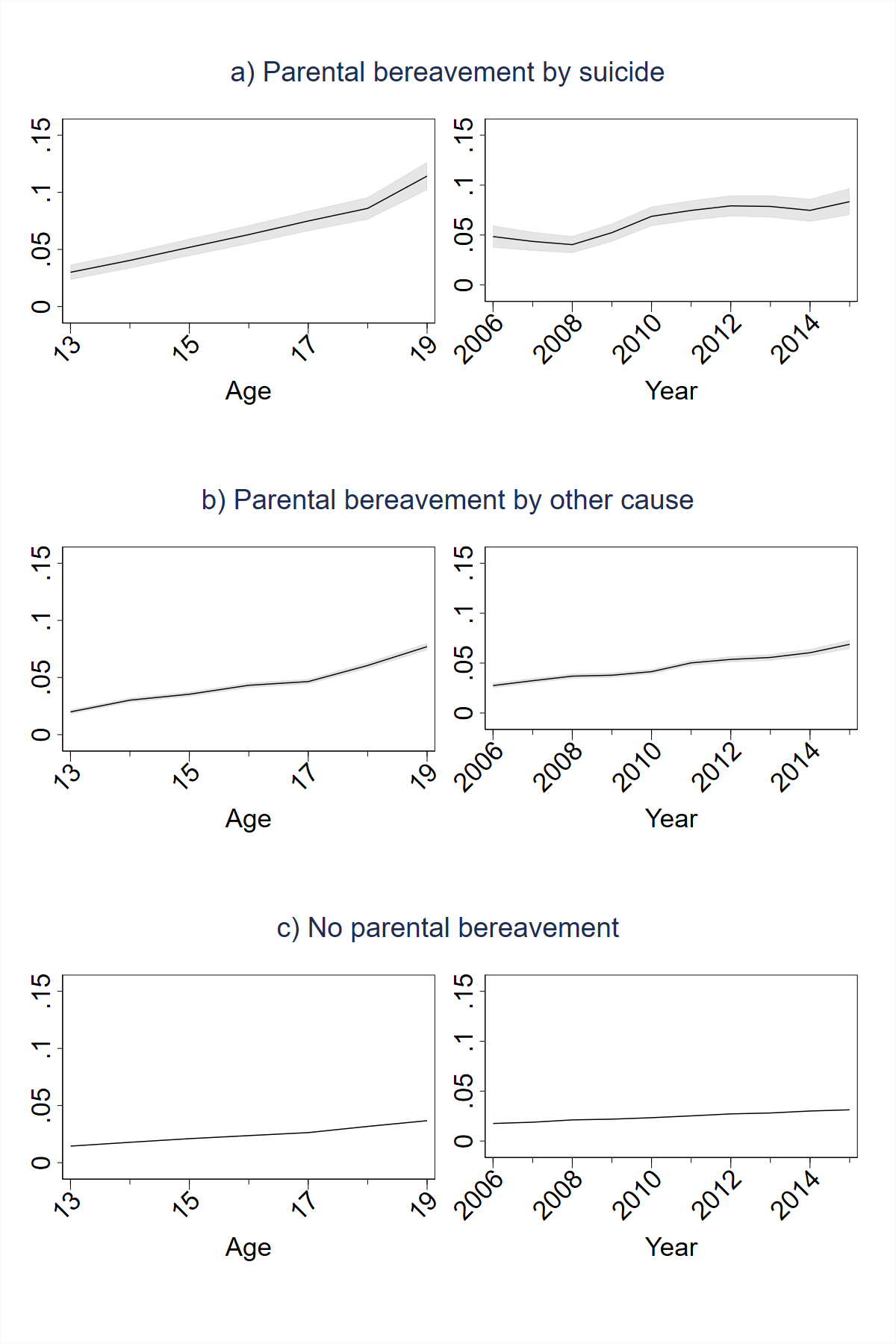
**

**Figure A.2. Alternate samples and outcomes.** See Table A.1 for parameter estimates and model info.


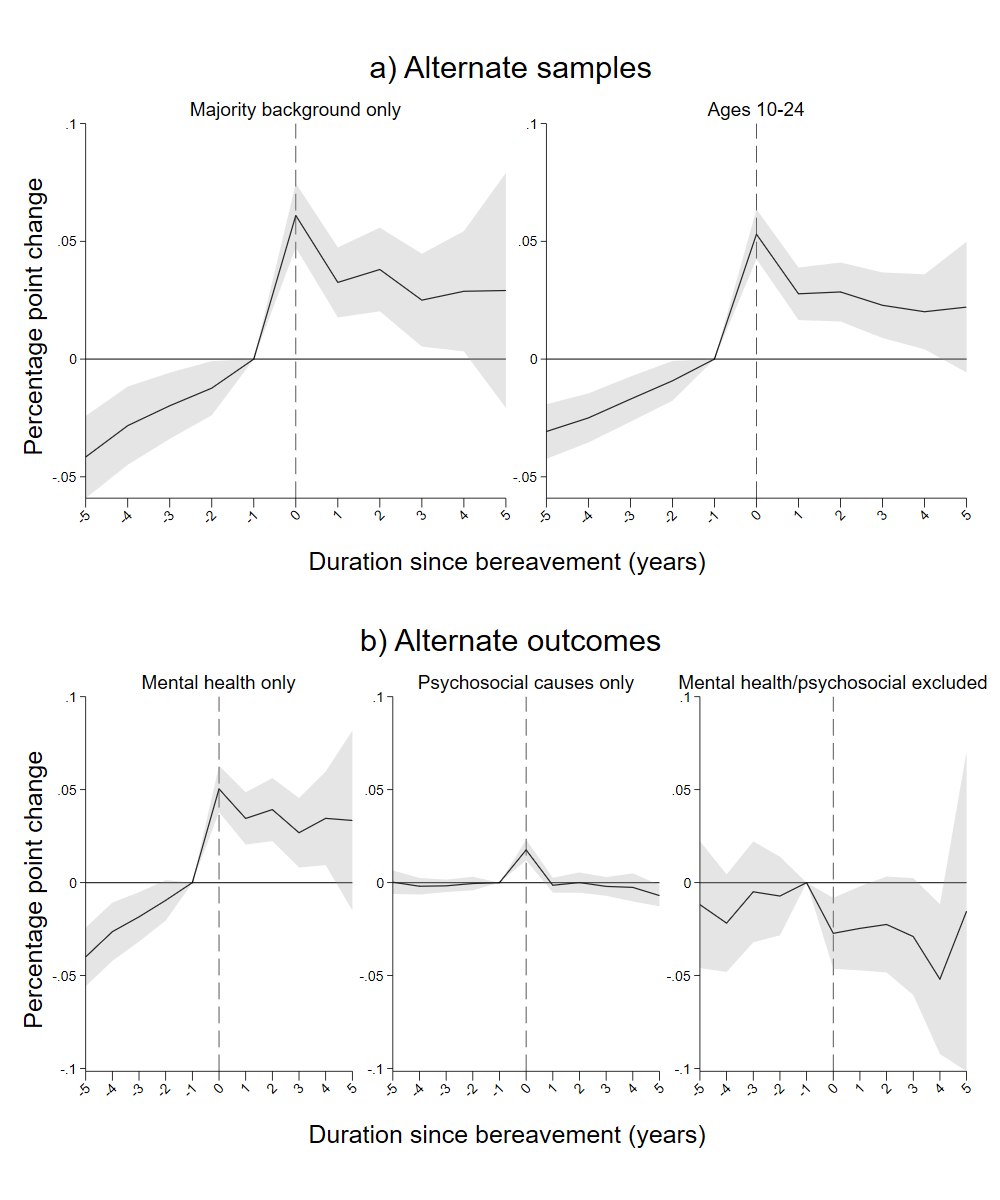


**Table A1. Models for alternate samples and outcomes.** Parameter estimates and standard errors. Estimates are controlled for dummies for age (one year categories), dummies for period time (one year categories), and individual fixed effects. Column 3 shows estimates for the interaction term between the stratifying variable and duration to bereavement. *** p<0.001, ** p<0.01, *p<0.05, +p<0.1.

| **A) Alternate samples (mental health and psychosocial consultations)** | | | | | |  |  |  |  |
| --- | --- | --- | --- | --- | --- | --- | --- | --- | --- |
| **Years from  bereavement** | **Majority background only** | | | **Ages 10-24** | | |  |  |  |
| -5 | -0.04 | (0.01) | *** | -0.03 | (0.01) | *** |  |  |  |
| -4 | -0.03 | (0.01) | *** | -0.02 | (0.01) | *** |  |  |  |
| -3 | -0.02 | (0.01) | ** | -0.02 | (0.00) | *** |  |  |  |
| -2 | -0.01 | (0.01) | * | -0.01 | (0.00) | * |  |  |  |
| -1 (ref.) | . | . |  |  |  |  |  |  |  |
| 0 | 0.06 | (0.01) | *** | 0.05 | (0.01) | *** |  |  |  |
| 1 | 0.03 | (0.01) | *** | 0.03 | (0.01) | *** |  |  |  |
| 2 | 0.04 | (0.01) | *** | 0.03 | (0.01) | *** |  |  |  |
| 3 | 0.03 | (0.01) | * | 0.02 | (0.01) | ** |  |  |  |
| 4 | 0.03 | (0.01) | * | 0.02 | (0.01) | * |  |  |  |
| 5 | 0.03 | (0.03) |  | 0.02 | (0.01) |  |  |  |  |
| N | 15 580 140 | |  | 25 150 916 | |  |  |  |  |
| **B) Alternate outcomes (main sample)** | | |  |  |  |  |  |  |  |
| **Years from  bereavement** | **Mental health only** | | | **Psychosocial causes only** | | | **Mental health/psychosocial excluded** | | |
| -5 | -0.04 | (0.01) | *** | 0.00 | (0.00) |  | -0.01 | (0.02) |  |
| -4 | -0.03 | (0.01) | *** | 0.00 | (0.00) |  | -0.02 | (0.01) |  |
| -3 | -0.02 | (0.01) | ** | 0.00 | (0.00) |  | 0.00 | (0.01) |  |
| -2 | -0.01 | (0.01) | + | 0.00 | (0.00) |  | -0.01 | (0.01) |  |
| -1 (ref.) | . | . |  | . | . |  |  |  |  |
| 0 | 0.05 | (0.01) | *** | 0.02 | (0.00) | *** | -0.03 | (0.01) | ** |
| 1 | 0.03 | (0.01) | *** | 0.00 | (0.00) |  | -0.02 | (0.01) | * |
| 2 | 0.04 | (0.01) | *** | 0.00 | (0.00) |  | -0.02 | (0.01) | + |
| 3 | 0.03 | (0.01) | ** | 0.00 | (0.00) |  | -0.03 | (0.02) | + |
| 4 | 0.03 | (0.01) | ** | 0.00 | (0.00) |  | -0.05 | (0.02) | * |
| 5 | 0.03 | (0.02) |  | -0.01 | (0.00) | * | -0.02 | (0.04) |  |
| N | 17 607 502 | |  | 17 607 502 | |  | 17 607 502 | |  |
